# Evaluation of pharmacists’ opioid dispensing practices: a cross-sectional study from Pakistan

**DOI:** 10.1080/20523211.2025.2557874

**Published:** 2025-11-05

**Authors:** Hafsa Arshad, Ali Hassan Gillani, Muhammad Arshed, Yu Fang

**Affiliations:** a Department of Pharmacy Administration and Clinical Pharmacy, School of Pharmacy, Xi’an Jiaotong University, Xi’an, People’s Republic of China; b Center for Drug Safety and Policy Research, Xian Jiaotong University, Xi’an, People’s Republic of China; c Shaanxi Center for Health Reform and Development Research, Xian, Shaanxi, People’s Republic of China; d Faculty of Allied Health Sciences, University Institute of Public Health, The University of Lahore, Lahore, Pakistan

**Keywords:** Pharmacist, opioid dispensing, opioids stewardship interventions, Punjab, Pakistan

## Abstract

**Backgrounds:**

Worldwide, the opioid crisis is escalating, and pharmacists are well-positioned to address opioid abuse. The objective of the study was to assess pharmacists’ knowledge, dispensing behaviours, concerns about physicians prescribing, provision of interventions to patients, and obstacles associated with opioid stewardship interventions.

**Methods:**

We employed a cross-sectional study design utilising a convenience sampling strategy to collect data from pharmacists employed in community and hospital pharmacies. A self-administered questionnaire of 58 items was utilised to gather data about pharmacists’ knowledge of opioids, dispensing procedures, and issues related to physicians’ prescribing behaviour from five cities of Punjab, Pakistan. Descriptive statistics were used for nominal and continuous variables. The Spearman Rho correlation was used to evaluate the correlation between knowledge, practice, and concern scores, while ANOVA was implemented to analyse the association between scores and demographics.

**Results:**

A total of 496 pharmacists responded, with a response rate of 72%. About 25% pharmacists were aware of using naloxone in opioid poisoning, 88.9% were aware of the potential risks and adverse effects of opioid therapy, and 87.5% explained these risks to patients. Almost half (48.4%) were concerned about physicians prescribing opioids to patients who were suspected of opioid misuse, and 64.5% were concerned that physicians prescribed opioids to patients who did not need them. The highest intervention provided by pharmacists was educating patients on safe and efficacious use of opioids (90.3% provided), and the least was recommendation of Naloxone in case of overdose (29% never provided). Almost 3/5th (58.0%) said lack of access to education or training resources was a high-impact barrier in opioid stewardship intervention provision.

**Conclusion:**

Pharmacists are concerned about physicians prescribing and mostly provide opioid-related training and interventions, but they also mentioned barriers to the provision of interventions. System-wide strategies are needed to improve opioid prescribing and physician-pharmacist communication.

## 1. Background

Pain alleviation constitutes a fundamental human right, although achieving effective pain management increasingly stresses the healthcare systems (HCS) of several countries (International-Pain-Summit, 2011; Mills et al., 2019). Opioid analgesics are often employed to manage moderate to severe pain; nevertheless, due to their significant potential for dependency, their prescribing and dispensing are meticulously regulated (Gregory & Gregory, 2020; Jantarada et al., 2021). Pertaining to the inconsistencies in the prescribing and dispensing practices, there is an escalation of global ‘Opioid Crisis’ (Spencer et al., 2024). The opioid crisis denotes the concerning rise in opioid-related overdose fatalities and opioid use disorder, principally attributed to the growing abuse of opioids (Mubarak et al., 2023). Annually, approximately 100,000 individuals submit to drug overdoses, with opioids accounting for 75% of these fatalities (Substance Abuse and Mental Health, 2023). In 2017, US President Donald Trump designated the opioid epidemic as a ‘Public Health Emergency,’ marking it as a significant drug catastrophe in American history (Mercia, 2017).

Opioids are placed in Schedule G of the Pakistan Punjab Drug Rules 2017 and also under the roof of High Alert Medications (HAM) in the guidelines given by the Drug Regulatory Authority Pakistan (DRAP) (Bashir et al., 2021; Salman et al., 2022). These guidelines properly demonstrate the prescribing and dispensing protocols, i.e. authorised physicians should prescribe narcotics and mention opiate use protocols in place of practice and adhere to precautions while prescribing (Arshad et al., 2025; Salman et al., 2022). Similarly, dispensing should also be regulated accordingly, i.e. a proper documentation, including the details of the patient, prescriber, and the exact dose of narcotic, should be registered by a competent pharmacist (Mubarak et al., 2023; Salman et al., 2022; Spencer et al., 2024). Pakistan faces lack of effective implementation of regulations, leading to the distribution of prescription-only drugs (Gillani et al., 2024; Majid Aziz et al., 2021; Malik, 2024; Malik et al., 2021), including opioids, from pharmacies without legitimate prescriptions (Bashir et al., 2021; Iqbal et al., 2021) and there is an increase in the countrywide opioid consumption rate between 2009 and 2019 in morphine milligram equivalents (MME) per 1000 inhabitants per day, which was 10.37 (Jayawardana et al., 2021). In Pakistan, the opioid crisis is also thought to have been exacerbated by the improper administration or poor handling of opioids in hospital or community settings and a lack of proper opioid stewardship programme (OSP) (Arshad et al., 2025; Iqbal et al., 2021, 2023; Islam, 2020; Jayawardana et al., 2021; Mubarak et al., 2023). OSP adheres to the same principles as antibiotic stewardship: ‘appropriate medication use for the appropriate patient at the appropriate time’ (Uritsky et al., 2020). Stewardship operates on the premise of optimising medication use achievable by improving prescribing practices, customising prescriber behaviours and expertise, and integrating shared decision-making within the HCS (Shrestha et al., 2023). Pharmacists are the cornerstone for the structured implementation of OSP.

Pharmacists, because of their frequent interactions with patients, may play a vital role in avoiding and resolving the opioid crisis. Pharmacists possess many opportunities to effectively educate patients on prevention and to promote the correct utilisation of opioid medications (Iqbal et al., 2022; Jadhari et al., 2024; Shrestha et al., 2024). Pharmacists can significantly contribute to the regulation of opioid distribution and diversion by meticulously overseeing opioid refills, preventing individuals from acquiring opioids from pharmacies, mitigating inappropriate opioid use in patients with potential for abuse, and monitoring non-medical utilisation of prescription opioids (Alvin et al., 2021; Webb et al., 2021). The readiness of pharmacists to address the opioid epidemic must be prioritised; yet, there is currently just one research from Pakistan assessing pharmacists’ abilities in addressing the opioid crisis (Mubarak et al., 2023). In Pakistan, pharmacists complete a five-year Doctor of Pharmacy (Pharm.D) degree, encompassing both theoretical and practical training in pharmacology, clinical pharmacy, and pharmacy practice (Mubarak et al., 2023, 2024). Opioid distribution procedures are frequently affected by regulatory deficiencies, insufficient training in pain treatment, and cultural attitudes towards opioid consumption (Arshad et al., 2025). In contrast to several high-income nations where pharmacists undergo specific training in the administration of controlled substances, Pakistani pharmacists frequently depend on experience and informal mentorship owing to the scarcity of continuing education programmes focused on opioid stewardship (Mubarak et al., 2023, 2024). It is essential to ascertain pharmacists’ apprehensions regarding patients’ opioid use, their encounters with opioid intoxication within the pharmacy, their worries pertaining to physicians’ opioid prescriptions, and the extent of intervention implemented to enhance the safety of opioid utilisation. The objective of the study was to assess pharmacists’ knowledge and attitudes towards opioids and stewardship, their dispensing behaviours, concerns regarding physicians’ opioid prescriptions, and their practices related to opioid interventions and barriers to opioid stewardship.

## 2. Methods

### 2.1. Study area and population

Pakistan is a country comprising four provinces (Punjab, Baluchistan, Sindh, and Khyber Pakhtunkhwa) and two autonomous administrative territories (Gilgit-Baltistan and Azad Jammu & Kashmir). Punjab constitutes around 26% of Pakistan's total area, although it is the most densely inhabited province, housing 60% of the country's inhabitants (Pakistan Bureau of Statistics, 2021). It was predicted that there were 23,029 licensed pharmacists in Punjab (Iqbal et al., 2021; Punjab Pharmacy Council, 2023). Pharmacists operate at many professional tiers, dependent upon the organisation, sector, objectives, and services provided. Each possesses a particular domain with specific obligations. The primary environments include community pharmacies, industrial sectors, and hospitals (Punjab Pharmacy Council, 2023). The HCS in Pakistan has three layers, with basic health units and rural health centres being the foundation of primary healthcare. Secondary care is delivered by tehsil and district headquarters, encompassing first- and second-referral facilities, whereas tertiary care is supplied by educational institutions (Khan et al., 2023). Pharmacists were included in the HCS of Pakistan and designated certain roles (procurement pharmacist, hospital pharmacist, clinical pharmacist, etc.) throughout various healthcare institutions (secondary and tertiary). We obtained data from pharmacists working in government hospitals, private hospitals, and community pharmacies.

### 2.2. Study design and data collection

This cross-sectional study gathered data from community and hospital pharmacists throughout five administrative divisions of Punjab, Pakistan from January 2022 to July 2022 using a structured paper-based questionnaire. We conveniently contacted pharmacies across five cities in Punjab (Lahore, Sahiwal, Rawalpindi, Sialkot and Faisalabad) and asked about the voluntary participation of pharmacists in the study. In Pakistan, certain chain pharmacies operate around the clock, with pharmacists rotating their shifts throughout the hours. We visited the pharmacies at various intervals to enhance pharmacist engagement. We approached the hospital pharmacist during working hours and obtained authorisation from hospital officials before data collection. We conveniently targeted 188 available pharmacists during the visits to hospitals, and those who indicated their willingness were given the paper-based questionnaire. The selection of hospitals was random, encompassing public and private hospitals and data were gathered by four teams of data collectors, primarily composed of pharmacy students, who were educated to adhere to protocols during data collection. Before the commencement of data collection, a cover letter was distributed to participants who consented to take part. The cover letter conveyed the necessity of preserving anonymity in their responses, underscored the voluntary aspect of their participation, and elucidated the purpose of our study to the pharmacist. Pharmacists were requested to endorse the letter by signing and providing their approval of the participation. We requested that participants complete the questionnaire on-site. A questionnaire was deemed useful if it was fully completed and contained a singular response for each question, necessitating one answer. To conduct a comprehensive assessment of data completeness, AHG independently verified and entered the data into Microsoft Excel, which was subsequently cross-checked by HA. No such incentive in any form has been provided to the pharmacist to participate in the study.

### 2.3. Study tool, validity and reliability

Individuals who participated in our research were provided with a survey instrument that was anonymous, self-administered, and consisted of 58 items, and it was divided into seven sections. An examination of the existing literature served as the basis for the development of this survey instrument (Kahan et al., 2011). The contents were comparable to the domains and structure of the physician survey that had been carried out in the past (Hutchinson et al., 2007; Morley-Forster et al., 2003; Nwokeji et al., 2007; Wenghofer et al., 2011), which we further simplified through discussions. The primary purpose of this form is to record the pharmacist's understanding of opioids and opioid stewardship, encounter the number of patients who have been prescribed opioids, the pharmacist's practices and behaviours surrounding opioid distribution, and the pharmacist's concerns regarding the opioid prescribing practices of physicians. We also examined the extent to which pharmacists experienced anomalous drug-related behaviour in patients, the interventions they provided during practice, and the impact of deterrents that prevented them from providing opioid stewardship interventions based on their experiences.

The first section of the questionnaire had information regarding demographics (gender, years of practice, age, medical speciality, and practice location). The second component consisted of 11 questions pertaining to the understanding of opioids, opioid stewardship, with the responses recorded in binary format (Yes/No). Section 3 consists of one question regarding the frequency of prescription with opioids and 9 questions related to practices of pharmacists regarding the provision of information for opioid management (Yes/No). Section 4 includes 6 questions which showed items regarding the concern about the opioid prescribing by physicians (Not at all concerned, a little concerned, somewhat, and very concerned). Further, this questionnaire surveyed pharmacists’ encounters with unusual drug-related behaviour observed in patients by asking 6 questions (Frequently, sometimes and not at all). A total of 7 items were present in the Section 6, which shows opioid stewardship interventions that pharmacists provide to patients during their practice using a 3-point scale (never provided, sometimes provided, always provided). The final section overhauls information on pharmacist perceptions of the impact of barriers to providing opioid stewardship interventions (5-point Likert very low, low, moderate, high and very high). The questionnaire is available in the Supplemental Material.

The concept and content of the questionnaire were validated by an extensive review of the relevant literature. The face validity of the data collection instrument was established by the evaluations of expert researchers who reviewed the items included in the instrument. Two faculty members with prior experience in pharmacy reviewed and assessed the questionnaire. Modifications were implemented to the instrument in accordance with the feedback provided by experts. Before commencing the research, the instrument underwent pilot testing to assess the comprehensibility and clarity of its components. Using pre-testing, we determined the respondents’ willingness to participate in the research and furnish the necessary information. The phrasing and scale were both clarified by making some small adjustments based on their suggestions. The internal consistency of the questionnaire was assessed using Cronbach’s alpha. Cronbach's alpha of the questionnaire was 0.81, indicating that the reliability of the study instrument is very good.

### 2.4. Statistical analysis

For analysing the demographics and other characteristics present in each of the parts, descriptive statistics were carried out. The mean and standard deviation were utilised for the representation of continuous variables. A record of the replies to each question in the section on knowledge, practices, drug-related behaviour, and concerns has been made, and these responses have been represented as numerical values and percentages. For determining the nature of the correlation that exists between the knowledge, practices, and concern sections, we utilised Spearman's Rho correlation test. We translated the knowledge (1–11) into scores by assigning a value of 1 to the correct answer and a value of 0 to the incorrect response. We collected all of the responses and assigned a value of one to each good practice and a value of zero to each negative practice (2–10), i.e. for question one, if the pharmacist asked about the patient’s pain level and medical history before dispensing opioids, then it showed appropriate practices and was numbered as 1. Similarly, the concern section (items 1–6) has been converted into scores by assigning a number to each response. For instance, a response indicating very concerned is assigned a score of 4, somewhat concerned is assigned a score of 3, a little concern is assigned a score of 2 and no concern at all is assigned a score of 1 (Ranging from 6 to 24). The aforementioned scores were aggregated and utilised in correlation. All the data were analysed using SPSS version 23.

## 3. Results

### 3.1. Demographic details

Out of 501 community pharmacists, 357 completed the survey, resulting in a response rate of 71.2%. Among hospital pharmacists, the response rate was 73.9% (139 out of 188). The diminished response rate may be ascribed to the substantial number of clients in community pharmacies and the considerable effort faced by pharmacists in hospital environments. The mean age of the pharmacists was 34.8 years (standard deviation 5.8 years). The majority of participants in our study were males with a mean age of 35.4 ± 5.4 years. The predominant proportion of pharmacists possessed a Pharm-D (48.4%), whereas 19.4% attained a PhD degree. The bulk of the targeted population was employed in private pharmacy settings (72%), with 44.8% working in chain pharmacies and 27.2% in other pharmacies. Furthermore, 28% of the participants worked in hospital environments, as specified in Table 1.

**Table 1.** Demographic information of 486 pharmacists from Punjab, Pakistan (Data from 2022).

**Gender**	***N* (%)**
Male	416 (83.9)
Female	80 (16.1)
**Practice experience**	***N* (%)**
1–5 years	176 (35.5)
5–10 years	128 (25.8)
> 10 years	192 (38.7)
**Education**	***N* (%)**
Pharm-D	240 (48.4)
M.Phil.	160 (32.3)
Ph.D.	96 (19.4)
**Age** (34.8 ± 5.8)	***N* (%)**
<25 years	32 (6.5)
25–35 years	256 (51.6)
36–45 years	208 (41.9)
**Practice setting**	***N* (%)**
Community pharmacy	222 (44.8)
Community chain pharmacy	135 (27.2)
Hospital setting	139 (28.0)

### 3.2. Knowledge about opioids and stewardship

The respondents exhibited the greatest awareness of potential hazards and adverse events associated with opioid therapy, followed by understanding of opioids, and knowledge that opioids are categorised under Schedule G of the Punjab Drug Rules 2007. Concerning guidelines and recommendations, just over two-thirds were acquainted with the WHO analgesic pain ladder, while just over half were informed of CDC guidelines for chronic pain. The minimal reaction was observed in the management of opioid poisoning, i.e. awareness of Naloxone use in overdose situations. In response to inquiries on opioid stewardship, 71.6% expressed awareness of the idea, while 70.8% acknowledged its potential to reduce the risk of opioid addiction and abuse (Table 2). The conversion of knowledge items into scores revealed a range from a minimum of 2 to a maximum of 11, with a mean score of 7.8 and a standard deviation of 1.5.

**Table 2.** Knowledge of 486 community and hospital pharmacists regarding opioids and opioid stewardship from Punjab, Pakistan (Data from 2022).

No.	Questions	Responses	
		Yes *N* (%)	No *N* (%)
1.	Do you know about opioid stewardship? (Opioid stewardship refers to a series of strategies and interventions involving the appropriate procurement, storage, prescribing and use of opioids, as well as the disposal of unused opioids when opioids are appropriately prescribed for the treatment and management of specific medical conditions)	355 (71.6)	141 (28.4)
2.	Do you know about opioid drugs?	437(88.1)	59 (11.9)
3.	Do you know about the WHO analgesic ladder of pain management?	340 (68.5)	156 (31.5)
4.	Do you know about the CDC Guideline for Prescribing Opioids for chronic pain?	276 (55.6)	220 (44.4)
5.	Are you aware of the potential risks and adverse effects associated with opioid therapy?	441 (88.9)	55 (11.1)
6.	Do you know that opioid drugs are included in Schedule G of the Punjab Drug Rules 2007?	416 (83.9)	16 (16.1)
7.	Do you know that opioid stewardship programmes can help reduce the risk of opioid addiction and abuse?	351 (70.8)	145(29.2)
8.	Do you know about guidelines and recommendations for prescribing opioids in chronic pain management	368 (74.2)	128 (25.8)
9.	Do you have adequate knowledge of the appropriate use of naloxone in opioid overdose situations?	127 (25.6)	369 (74.4)
10.	Are you familiar with the risk factors for opioid abuse and addiction?	400 (80.6)	96 (19.4)
11.	Are you aware of the resources available for patients who require assistance with opioid tapering or addiction treatment?	304 (61.3)	192 (38.7)

### 3.3. Dispensing practices to patients

**Figure 1.**
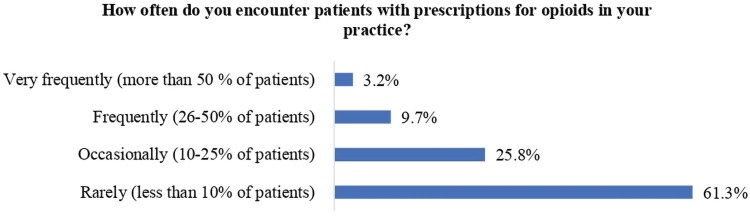
Frequency of opioid prescription encounters by pharmacists.

Upon the query of the number of encounters with patients carrying the prescription for opioids large population of pharmacists demonstrated rare encounters, and only 3.2% of them have very frequent encounters with opioid prescriptions. The details are given in Figure 1.

The majority of pharmacists in our investigation demonstrated proficient practices concerning the safe use of opioids. A large proportion of pharmacists inquired about patients’ pain levels and medical histories, guided on the storage requirements for opioids, advised patients on proper utilisation of opioids, addressed the adverse effects and risks associated with opioids, and mentioned the potential for opioid addiction and dependency (Table 3). Upon converting the questions into scores, a significant variety in answers was observed, ranging from 0 to 9. The average overall practice score was 7.7 ± 1.9.

**Table 3.** Dispensing practices of 486 community and hospital pharmacists regarding safe use of opioids from Punjab, Pakistan (Data from 2022).

No.	Statements	Yes	No
		*N* (%)	*N* (%)
1.	Do you ask about the patient’s pain level and medical history before dispensing opioids?	416 (83.9)	80 (16.1)
2.	Do you provide counselling on the safe use of opioids before dispensing?	427 (86.1)	69 (13.9)
3.	Do you explain the potential side effects and risks associated with opioids?	434 (87.5)	62 (12.5)
4.	Do you verify the prescription before dispensing opioids?	416 (83.9)	80 (16.1)
5.	Do you provide information on the proper storage and disposal of opioids?	448 (90.3)	48 (9.7)
6.	Do you discuss the risks of opioid addiction and dependence?	439 (88.5)	57 (11.5)
7.	Do you provide information on alternative treatments for pain management?	448 (90.3)	48 (9.7)
8.	Do you suggest non-opioid pain relievers or non-pharmacological treatments?	432 (87.1)	64 (12.9)
9.	Do you explain the importance of following the prescribed dose and not exceeding it?	464 (93.5)	32 (6.5)

### 3.4. Pharmacists’ concerns about physicians’ prescribing

Table 4 illustrates pharmacists’ apprehensions regarding physicians’ opioid prescriptions. Upon assessment, about 2/3rd of pharmacists expressed concern (somewhat and very concerned) about the co-prescription of benzodiazepines with opioids, 64.5% were concerned that physicians prescribed opioids to patients who likely did not require them, and about half were concerned about prescribing opioids to patients suspected of opioid misuse. The mean concern score was 17.5 ± 4.0, with scores ranging from 6 to 24.

**Table 4.** Pharmacists’ concerns about physicians’ opioid prescribing practices from Punjab, Pakistan.

No	Statement	Not at all concerned *N* (%)	A little concerned *N* (%)	Somewhat concerned *N* (%)	Very concerned *N* (%)
1	Physicians prescribed benzodiazepines along with opioids	32 (6.5)	128 (25.8)	160 (32.3)	176 (35.5)
2	Physicians prescribed opioids to patients you suspected of opioid misuse	16 (3.2)	240 (48.4)	112 (22.6)	128(25.8)
3	Physicians prescribed opioids to patients who, in your opinion, probably do not need them	16 (3.2)	160 (32.3)	112 (22.6)	208(41.9)
4	Physicians prescribed opioid doses that, in your opinion, might be too high	64 (12.9)	96 (19.4)	96 (19.4)	240 (48.4)
5	Physician prescribed opioids in combination with other NSAID drugs, e.g. acetaminophen, where the dose of the acetaminophen is too high	96 (19.4)	64 (12.9)	176 (35.5)	160 (32.3)
6	Physicians prescribed injectable opioids for chronic non-cancer pain	32 (6.5)	112 (22.6)	176 (35.5)	176 (35.5)

### 3.5. Spearman correlation between knowledge, practices and concerns

We employed Spearman correlation to examine the relationship between the pharmacist's knowledge, practices, and concerns scores. It revealed a highly significant positive connection between knowledge and practice, as well as between knowledge and concern scores. Nevertheless, a negligible inverse correlation seemed to exist between practice scores and concern scores of pharmacists. The detail is elucidated in Table 5.

**Table 5.** Spearman correlation between knowledge, practice and concerns score from Punjab, Pakistan (N 486; data from 2022).

Correlations					
			Knowledge score	Practices score	Concerns score
Spearman’s rho	Knowledge score	Correlation Coefficient	1.000	0.401**	0.267**
		Sig. (2-tailed)	.	0.000	0.000
	Practice score	Correlation Coefficient	0.401**	1.000	−0.034
		Sig. (2-tailed)	0.000	.	0.455
	Concerns score	Correlation Coefficient	.267**	−0.034	1.000
		Sig. (2-tailed)	0.000	0.455	.

**. Correlation is significant at the 0.01 level (2-tailed).

### 3.6. Patients unusual drug-related behaviour

**Figure 2.**
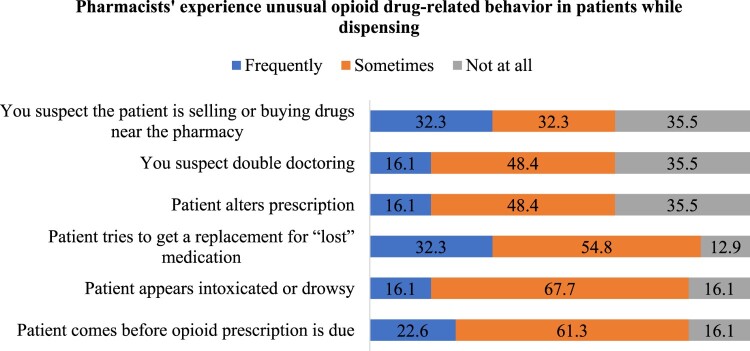
Pharmacists’ experiencing unusual drug-related behaviour in patients.

Behaviour indicative of opioid usage or dependence is referred to as aberrant drug-related behaviour. Figure 2 indicates that the most often described irregular conduct was patients obtaining replacements for lost drugs. The second most prevalent phenomenon included patients visiting pharmacies before the scheduled refill period. The third most prevalent deviant behaviour seen was individuals seeming drowsy at pharmacies (Figure 2).

### 3.7. Opioid stewardship interventions

The pharmacist provided interventions to the patients upon their contact during practices. A very large number of respondents educated patients on the judicious use of opioids suggested non-opioid pain management to the patient and recommended non-opioid pain management therapy to the provider. In case of opioid overdose management, 29.0% never recommended Naloxone to a patient at risk of opioid overdose (Table 6).

**Table 6.** Number of pharmacists providing interventions to patients during dispensing practice (*N* = 486).

No.	Opioid stewardship interventions	Never provided *N* (%)	Sometimes provided *N* (%)	Always provided *N* (%)
1.	Educated patients on safe use of opioids	48 (9.7)	192 (38.7)	256 (51.6)
2.	Consulted a prescription drug monitoring program before dispensing an opioid	96 (19.4)	208 (41.9)	192 (38.7)
3.	Recommended non-opioid pain management therapy for the patient	16 (3.2)	320 (64.5)	160 (32.3)
4.	Recommended or dispensed naloxone to the patient at risk of opioid overdose	144 (29.0)	288 (58.1)	64 (12.9)
5.	Educated patient on medication(s) for opioid use disorder	96 (19.4)	192 (38.7)	208 (41.9)
6.	Recommended non-opioid pain management therapy to the provider	96 (19.4)	224 (45.2)	176 (35.5)
7.	Referred patient to addiction recovery resource	128 (25.8)	192 (38.7)	176 (35.5)

### 3.8. Barriers to opioid stewardship interventions

The most significant patient-related issues identified as obstacles to the provision of treatments were patient refusal (35.5%: 25.8% high impact and 9.8% very high impact). Likewise, insufficient room for private conversations (48.4–32.3% high effect and 16.1% very high impact) and insufficiency of personnel and time (48.4–35.5% high impact and 12.9% very high impact) were identified as other obstructions related to the working environment. The information has been included in Table 7. Supplemental Material – Table S8 depicts the score association of demographics with the knowledge, practice and concerns score.

**Table 7.** Pharmacists’ perceptions of the impact of barriers to providing opioid stewardship interventions from Punjab, Pakistan (*N* = 486, Data from 2022).

No.	Barriers to providing opioid stewardship interventions	Very low impact *N* (%)	Low Impact *N* (%)	Moderate Impact *N* (%)	High impact *N* (%)	Very high impact *N* (%)
	**Patient interaction barriers**					
1.	Patient refusal	48 (9.7)	32 (6.5)	240 (48.4)	128 (25.8)	48 (9.7)
2.	Compromised pharmacist/patient relationship	16 (3.2)	80 (16.1)	240 (48.4)	112 (22.6)	48 (9.7)
3.	Negative patient reaction	16 (3.2)	64 (12.9)	288 (58.1)	64 (12.9)	64 (12.9)
4.	Compromised personal safety	16 (3.2)	48 (9.7)	224 (45.2)	160 (32.3)	48 (9.7)
	**Work environment barriers**					
5.	Minimal or no reimbursement for interventions	32 (6.5)	112 (22.6)	256 (51.6)	48 (9.7)	48 (9.7)
6.	Inadequate staffing or time to make interventions	48 (9.7)	80 (16.1)	128 (25.8)	176 (35.5)	64 (12.9)
7.	No space for private conversations	48 (9.7)	64 (12.9)	144 (29.0)	160 (32.3)	80 (16.1)
8.	Lack of management support	64 (12.9)	64 (12.9)	144 (29.0)	192 (38.7)	32 (6.5)
9.	Lack of colleague support	96 (19.4)	48 (9.7)	240 (48.4)	80 (16.1)	32 (6.5)
	**Confidence or knowledge barriers**					
10.	Low comfort or confidence in making interventions	64 (12.9)	64 (12.9)	128 (25.8)	192 (38.7)	48 (9.7)
11.	Lack of access to educational or training resources	48 (9.7)	48 (9.7)	112 (22.6)	208 (41.9)	80 (16.1)
12.	Lack of familiarity with interventions	48 (9.7)	48 (9.7)	176 (35.5)	144 (29.0)	80 (16.1)

## 4. Discussion

Despite the limited research on opioids, no previous studies from Pakistan have examined pharmacists’ knowledge, practices, concerns, and interventions during distribution, save for a study by Mubarak et al. (2023) that assessed pharmacists’ abilities in opioid management. The opioid distribution issue is concerning; yet, no prior activities have been implemented to yield evidence-based results that might guide strategies for decreasing prescription opioid consumption in Pakistan. Our research is, to the best of our knowledge, the inaugural study examining the pharmacist's role in mitigating opioid misuse and abuse within the drug use system.

### 4.1. Pharmacist opioid-related knowledge and practices

Pharmacists in our sample had a sufficient comprehension of the CDC guidelines and the WHO analgesic ladder. The results closely resembled those of a Canadian study, where half the population acknowledged the national recommendations for opioid use and therapy (Patel et al., 2016), and those from the USA, where almost 3/4th of pharmacists were aware of the WHO pain ladder (Kosobuski et al., 2022). The WHO analgesic ladder, mostly utilised for cancer pain, has demonstrated efficacy in pain management and has repeatedly been recognised as a crucial strategy for opioid stewardship and addiction prevention (Nicholson, 2003; Vargas-Schaffer, 2010). Pharmacists had little comprehension of opioid overdose therapy and Naloxone delivery in poisoning scenarios; a similar trend was observed in the USA, where a small proportion of pharmacists felt competent to dispense Naloxone without a prescription (Thornton et al., 2017). Pharmacists’ training must encompass the safe administration of Naloxone, since it is essential in combating the opioid crisis and be made compulsory to prepare pharmacists for poisoning management, since data demonstrate that pharmacist-led treatments reduce fatality rates (Bach & Hartung, 2019; Leong et al., 2016; Saldana et al., 2017; Skoy et al., 2022).

Pharmacists in our study were keen to guide the appropriate storage and disposal of opioids, consistent with previous studies (Skoy et al., 2022; Thomson et al., 2019). Likewise, our group provided the danger of drug treatment side effects, a phenomenon previously seen in studies (Skoy et al., 2022; Thakur et al., 2019). Pharmacists may contribute to the prevention of opioid-related harm by educating patients on the negative effects of opioid medications, particularly those with potential for habit building (Compton et al., 2019; Skoy et al., 2022). Pharmacists, being the last healthcare professionals (HCPs) consumers interact with before utilising drugs, are well situated to detect diversion, oversee potentially hazardous usage of prescription opioids, and inform patients about opioid-related hazards (Bach & Hartung, 2019).

### 4.2. Pharmacists’ concern

A significant percentage of pharmacists expressed concerns with physicians prescribing benzodiazepines in conjunction with opioids and administering opioids to those who were at elevated risk of opioid abuse. Comparable outcomes were seen in previous findings from Canada (Kahan et al., 2011). These problems are partially linked to the communication gap between pharmacists and physicians (Kahan et al., 2011). In a qualitative investigation, community pharmacists indicated significant challenges in contacting doctors, since receptionists often refused to transfer their calls (Hughes & McCann, 2003). In another qualitative research, 27 pharmacists and 36 general practitioners documented the nature of their interprofessional interactions in diaries. Some of the interactions (57%) were made to elucidate prescription information, while just 4% pertained to patient care (Weissenborn et al., 2017), indicating that pharmacists and doctors seldom engage in discussions about clinical matters. Adopting secure protocols for prescription opioids can significantly mitigate these risks. Furthermore, it will limit the accessibility of diverted opioids, since the volume of diverted opioids is strongly correlated with the volume of prescription opioids. Common strategies employed in OSP encompass advocating for the use of adjunct non-opioid analgesics, such as paracetamol and nonsteroidal anti-inflammatory drugs, restricting opioid prescribing quantities, integrating pharmacist consultation on proper usage, management, and disposal of opioids, and instructing junior staff and other prescribers (Australian Commission, 2021). The efficacy of the OSP depends on the cooperation of leadership, the team, and vital support groups. The makeup of team members may vary depending on the institution and the resources available (Ha et al., 2019). A positive linear association exists between the knowledge score and the concern score. Prior studies have shown that a constructive comprehension yields desired outcomes (Gillani et al., 2019).

### 4.3. Opioid-related interventions and barriers to the provision of interventions

A significant number of patients were instructed on the prudent use of opioids and informed about the medications for opioid use disorder. A significant proportion recommended non-opioid pain therapy to both patients and providers. Comparable results were seen in a trial conducted in the USA, where pharmacists administered one or more risk-factor-dependent interventions to 41.1% of the participants (Skoy et al., 2022). Previous studies indicate that community pharmacists have a robust interest in assisting patients with opioid use disorder and possess positive opinions towards pharmacy-based screening and intervention initiatives (Cochran et al., 2013, 2015). Evidence indicates that patients are amenable to concise educational interventions about opioid safety and prevention of overdose when it is administered by pharmacists in the emergency department (Winstanley et al., 2017). The screening and short intervention models, with or without treatment referral, are evidence-based strategies aimed at identifying and mitigating risks linked to problematic drug and alcohol use. The concept has been successfully used in many clinical and community settings and is effective (Bach & Hartung, 2019). The implementation of screening, brief intervention by community pharmacists has been evaluated in different settings. Such approaches should be used in the Pakistani context to reduce opioid usage. However, additional models, such as routine opioid outcome monitoring and Motivational Intervention–Medication Therapy Management, should also be utilizsed within our HCS to assess their effectiveness in addressing the opioid crisis (Cochran et al., 2018; Nielsen et al., 2020).

Among the obstacles to intervention, two in five respondents identified insufficient management support as a significant barrier, while one in five noted inadequate colleague support and limited reimbursement for interventions as considerable impediments to their implementation. These findings align with the research by Vadiei et al., which identified substantial obstacles such as inadequate collaboration among HCPs and patients, unwelcoming interactions with prescribers, insufficient reimbursement for services, lack of corporate support, and time limitations (Vadiei et al., 2022). Improved education and training in pharmacy services, together with corporate and organisational backing, would enhance pharmacists’ ability and confidence to tackle the opioid crisis (Patel et al., 2016; Thakur et al., 2019).

## 5. Implication for policy, practice and future prospects

1. This study provided empirical evidence on different aspects of pharmacist-led opioid dispensing and opioid stewardship interventions, highlighting critical gaps in existing literature which can serve as a foundation for future research to assess long-term impact of pharmacists’ opioid dispensing interventions and their barriers to tackle the opioid crisis.
2. The study highlighted the opioid stewardship intervention barriers while opioid dispensing enhanced the need for training programmes, resources equipped to skill pharmacists to combat opioid related challenges, allowing them to act as frontline healthcare providers to combat the opioid crisis.
3. It facilitates the execution of standardised opioid dispensing procedures, such as pain management screening instruments, counselling guidelines, and integrated patient care monitoring programmes and interventions within multidisciplinary healthcare environments.
4. The PharmD curriculum must be revised to provide comprehensive coverage of opioid use disorder, misuse, and diversion at both graduate and undergraduate levels. Pharmacology, clinical, and forensic pharmacy may encompass a comprehensive opioid use curriculum. Delphi, nominal group, and other consensus methodologies can ascertain module components.
5. Future research should concentrate on legislation and strategies to guarantee the involvement of community pharmacists. Research is essential to develop evidence-based opioid stewardship interventions, clinical support instruments, and collaborative practice frameworks to enhance communication among pharmacists, prescribers, and regulatory agencies.

### 5.1. Strengths and limitations

This study is unprecedented since it is the first to assess opioid-related attitudes, interventions, and barriers to intervention across several contexts in Punjab, Pakistan. This study serves as a foundation for the formulation or refinement of policies for the safe and prudent use of opioids. This is the initial evidence in Pakistan illustrating pharmacists’ worries about opioid prescription.

This research has limitations, including its confinement to a cross-sectional analysis and the lack of assessment of respondents’ interest in further exploring certain therapeutic subjects, such as the utilisation of addiction screening instruments. Future studies may address the aforementioned issues by validating the dependability of reported confidence levels in opioid prescriptions by a comparison with actual practice patterns. Conducting qualitative research will yield an in-depth understanding of pharmacists’ viewpoints about their apprehensions and the adverse experiences faced by patients. Secondly, the poll solely evaluated pharmacists’ subjective interactions with patients utilising opioids, depending on responders’ personal assessments of frequency through categories like ‘sometimes’ and ‘frequently.’ This may yield an exaggerated depiction of the prevalence and ramifications of addiction. A patient who exhausts their opioid supply early is not always engaging in abuse or addiction; also, certain pharmacists may overrate the prevalence of this behaviour. Finally, we took multiple pharmacists from the same setting, which would skew the findings and outcomes, and it cannot be generalisable across all settings.

## 6. Conclusion

Pharmacists commonly exhibit good opioid and stewardship-related knowledge and provide stewardship-related intervention. Pharmacists tackled aberrant drug-related behaviour in their patients, and they were concerned about physicians' prescribing patterns. These could be rectified by increasing the pharmacist-physician interaction regarding opioid therapy. These results further emphasise the need for a system-wide response, collaboration and implementation of OSP. Also, there should be implementation of national opioid prescribing guidelines, provincial-level prescribing databases, and strengthening of protocols for physician-pharmacist communication.

## Supplementary Material

Supplemental Material - Tables

Supplemental Material - Data Collection Tool

## Data Availability

The datasets used and/or analysed during the current study are available from the corresponding author on reasonable request.
