# Supplementary material for: Evaluation of pharmacists’ opioid dispensing practices: a cross-sectional study from Pakistan
Source: J Pharm Policy Pract. 2025 Nov 5;18(1):2557874. doi: 10.1080/20523211.2025.2557874 (PMC12590573; doi:10.1080/20523211.2025.2557874)
Supplement: Supplemental Material - Tables [file JPPP_A_2557874_SM1106.docx]

**Table8: Demographic association with Knowledge, attitude and concerns score (T test ANOVA)**

| **No** | **Demographic variable** | **Knowledge score (Mean±SD)** | **P** | **Attitude practice score (Mean±SD)** | **P** | **Concerns score (Mean±SD)** | **P** |
| --- | --- | --- | --- | --- | --- | --- | --- |
| **1** | **Gender** |  |  |  |  |  |  |
|  | Male | 7.7(1.5) | 0.002 | 7.9(1.9) | 0.000 | 17.5(4.1) | 0.72 |
|  | Female | 8.3(1.4) |  | 6.9(1.2) |  | 17.4(3.4) |  |
| **2** | **Age group (Years)** |  |  |  |  |  |  |
|  | <25 | 7.8(1.2) |  | 6.0(1.0) |  | 17.5(2.5) |  |
|  | 25-35 | 7.8(1.5) | 0.98 | 7.7(2.1) | 0.000 | 17.5(3.5) | 0.91 |
|  | 36-45 | 7.8(1.7) |  | 8.1(1.4) |  | 17.6(4.6) |  |
| **3** | **Education** |  |  |  |  |  |  |
|  | Pharm-D | 8.1(1.7) |  | 8.0(1.4) |  | 18.4(3.0) |  |
|  | MPhil | 7.4(1.3) | 0.000 | 7.3(2.6) | 0.001 | 17.8(3.8) | 0.000 |
|  | Ph.D | 7.6(1.5) |  | 8.0(1.5) |  | 15.0(5.3) |  |
| **4** | **Practice experience (Years)** |  |  |  |  |  |  |
|  | 1-5 | 7.5(1.5) |  | 7.0(2.6) |  | 19.1(2.8) |  |
|  | 6-10 | 7.6(1.6) | 0.000 | 7.7(1.3) | 0.000 | 16.4(3.7) | 0.000 |
|  | >10 | 8.2(1.6) |  | 8.5(1.1) |  | 16.9(4.7) |  |
| **5** | **Practice settings** |  |  |  |  |  |  |
|  | Community pharmacy | 7.8(1.6) |  | 8.3(1.3) |  | 17.7 (4.1) |  |
|  | Community chain pharmacy | 7.8(1.5) | 0.82 | 7.9(1.2) | 0.000 | 18.1(3.4) | 0.068 |
|  | Hospital setting | 7.7(1.8) |  | 6.9(2.9) |  | 17.1(4.9) |  |
|  |  |  |  |  |  |  |  |
