# Supplementary material for: Evaluation of pharmacists’ opioid dispensing practices: a cross-sectional study from Pakistan
Source: J Pharm Policy Pract. 2025 Nov 5;18(1):2557874. doi: 10.1080/20523211.2025.2557874 (PMC12590573; doi:10.1080/20523211.2025.2557874)
Supplement: Supplemental Material - Data Collection Tool [file JPPP_A_2557874_SM1105.docx]

**Pharmacist’s opioid dispensing practices, interaction with patients, and opioid stewardship interventions to patients during dispensing opioid therapy**

**Section 1: Demographic Information**

| 1. **Gender** | - Male - Female | **4. Age** | - <25 years - 25-35 years - 36-45 years - 46-55years - >55 years |
| --- | --- | --- | --- |
| 1. **Practice Experience** | - 1-5 years - 5-10 years - > 10 years | **5. Practice setting** | - Community pharmacy - Community chain pharmacy - Hospital clinical setting |
| 1. **Province** | - Punjab - Khyber Pakhtunkhwa - Sindh - Balochistan - Gilgit-Baltistan | **6. Education** | - Pharm-D - M.Phil - Ph.D |

**Section 2: Knowledge about opioid stewardship**

1. Do you feel you have adequate knowledge on opioid stewardship?

| **No.** | **Questions** | **Responses** | |
| --- | --- | --- | --- |
|  |  | **Yes** | **No** |
|  | Do you know about opioids stewardship?  (Opioid stewardship refers to a series of strategies and interventions involving the appropriate procurement, storage, prescribing and use of opioids, as well as the disposal of unused opioids when opioids are appropriately prescribed for the treatment and management of specific medical conditions) |  |  |
|  | Do you know about opioid drugs |  |  |
|  | Do you know about WHO analgesic ladder of pain management? |  |  |
|  | Do you know about the CDC Guideline for Prescribing Opioids for Chronic Pain? |  |  |
|  | Do you aware of the potential risks and adverse effects associated with opioid therapy. |  |  |
|  | Do you know opioid drugs are included in schedule G drugs by drug regulatory authority of Pakistan |  |  |
|  | Do you know opioid stewardship programs can help reduce the risk of opioid addiction and abuse? |  |  |
|  | Do you know about guidelines and recommendations for prescribing opioids in chronic pain management |  |  |
|  | Do you have adequate knowledge on the appropriate use of naloxone in opioid overdose situations? |  |  |
|  | Are you familiar with the risk factors for opioid abuse and addiction? |  |  |
|  | Do you aware of the resources available for patients who require assistance with opioid tapering or addiction treatment. |  |  |

**Section 3: Dispensing practice and attitude**

1. **How often do you encounter patients with prescriptions for opioids in your practice?**

- Rarely (less than 10% of patients)
- Occasionally (10-25% of patients)
- Frequently (26-50% of patients)
- Very frequently (more than 50 % of patients)

**Dispensing practice and attitude**

1. Do you ask about patient’s pain level and medical history before dispensing opioids? (Yes/No)
2. Do you provide counseling on the safe use of opioids before dispensing? (Yes/No)
3. Do you explain the potential side effects and risks associated with opioids? (Yes/No)
4. Do you verify the prescription before dispensing opioids? (Yes/No)
5. Do you provide information on the proper storage and disposal of opioids? (Yes/No)
6. Do you discuss the risks of opioid addiction and dependence? (Yes/No)
7. Do you provide information on alternative treatments for pain management? (Yes/No)
8. Do you suggest non-opioid pain relievers or non-pharmacological treatments? (Yes/No)
9. Do you explain the importance of following the prescribed dose and not exceeding it? (Yes/No)

**Section 4: Please rate the extent of pharmacist concerns about physicians’ opioid prescribing practices while dispensing**

| **No** | **Concerns about physician opioid prescribing practices while dispensing** | **Not at all**  **concerned** | **A little**  **concerned** | **Somewhat**  **concerned** | **Very**  **concerned** |
| --- | --- | --- | --- | --- | --- |
| **11** | Physician prescribed benzodiazepines along with opioids |  |  |  |  |
|  | Physician prescribed opioids to patients you suspect of opioid misuse |  |  |  |  |
|  | Physician prescribed opioids to patients who, in your opinion, probably do not need them |  |  |  |  |
|  | Physician prescribed opioid doses that, in your opinion, might be too high |  |  |  |  |
|  | Physician prescribed opioids in combination with other NSAID drugs e.g. acetaminophen where dose of the acetaminophen is too high |  |  |  |  |
|  | Physician prescribed injectable opioids for chronic non cancer pain |  |  |  |  |

**Section 5: Pharmacist’s experience unusual drug-related behavior observed in patients while dispensing**

| **No.** | **Unusual drug-related behavior while dispensing** | **Frequently** | **Sometimes** | **Not at all** |
| --- | --- | --- | --- | --- |
|  | Patient comes before opioid prescription is due |  |  |  |
|  | Patient appears intoxicated or drowsy |  |  |  |
|  | Patient tries to get a replacement for “lost” medication |  |  |  |
|  | Patient alters prescription |  |  |  |
|  | You suspect double doctoring |  |  |  |
|  | You suspect the patient is selling or buying drugs near the pharmacy |  |  |  |

**Section 6: Have you provided the following opioid stewardship interventions to patients during your practice?**

| **No.** | **Opioid stewardship interventions** | **Never provided** | **Sometimes provided** | **Always provided** |
| --- | --- | --- | --- | --- |
|  | Educated patients on safe use of opioids |  |  |  |
|  | Consulted a prescription drug monitoring program before dispensing an opioid |  |  |  |
|  | Recommended non-opioid pain management therapy to patient |  |  |  |
|  | Recommended or dispensed naloxone to patient at risk of opioid overdose |  |  |  |
|  | Educated patient on medication(s) for opioid use disorder |  |  |  |
|  | Recommended non-opioid pain management therapy to provider |  |  |  |
|  | Referred patient to addiction recovery resource |  |  |  |
|  | Used screening tool to identify opioid misuse or opioid use disorder |  |  |  |

**Section 7: Pharmacist perceptions of the impact of barriers to providing opioid stewardship interventions**

| **No.** | **Impact of barriers to providing opioid stewardship interventions** | **Very**  **low impact** | **Low**  **impact** | **Moderate**  **impact** | **High**  **impact** | **Very**  **high impact** |
| --- | --- | --- | --- | --- | --- | --- |
|  | **Patient interaction barriers** |  |  |  |  |  |
|  | Patient refusal |  |  |  |  |  |
|  | Compromised pharmacist/patient relationship |  |  |  |  |  |
|  | Negative patient reaction |  |  |  |  |  |
|  | Compromised personal safety |  |  |  |  |  |
|  | **Work environment barriers** |  |  |  |  |  |
|  | Minimal or no reimbursement for interventions |  |  |  |  |  |
|  | Inadequate staffing or time to make interventions |  |  |  |  |  |
|  | No space for private conversations |  |  |  |  |  |
|  | Lack of management support |  |  |  |  |  |
|  | Lack of colleague support |  |  |  |  |  |
|  | **Confidence or knowledge barriers** |  |  |  |  |  |
|  | Low comfort or confidence in making interventions |  |  |  |  |  |
|  | Lack of access to education or training resource |  |  |  |  |  |
|  | Lack of familiarity with interventions |  |  |  |  |  |

**Thanks for your time**
